# Supplementary material for: Ribosomal DNA copy number variation associates with hematological profiles and renal function in the UK Biobank
Source: Cell Genom. 2024 May 14;4(6):100562. doi: 10.1016/j.xgen.2024.100562 (PMC11228893; doi:10.1016/j.xgen.2024.100562)
Supplement: Document S1. Figures S1‒S23 [file mmc1.pdf]

Cell Genomics, Volume *4*

Supplemental information

Ribosomal DNA copy number variation associates with hematological profiles and renal function in the UK Biobank

Francisco Rodriguez-Algarra, David M. Evans, and Vardhman K. Rakyan

Supplemental Figures

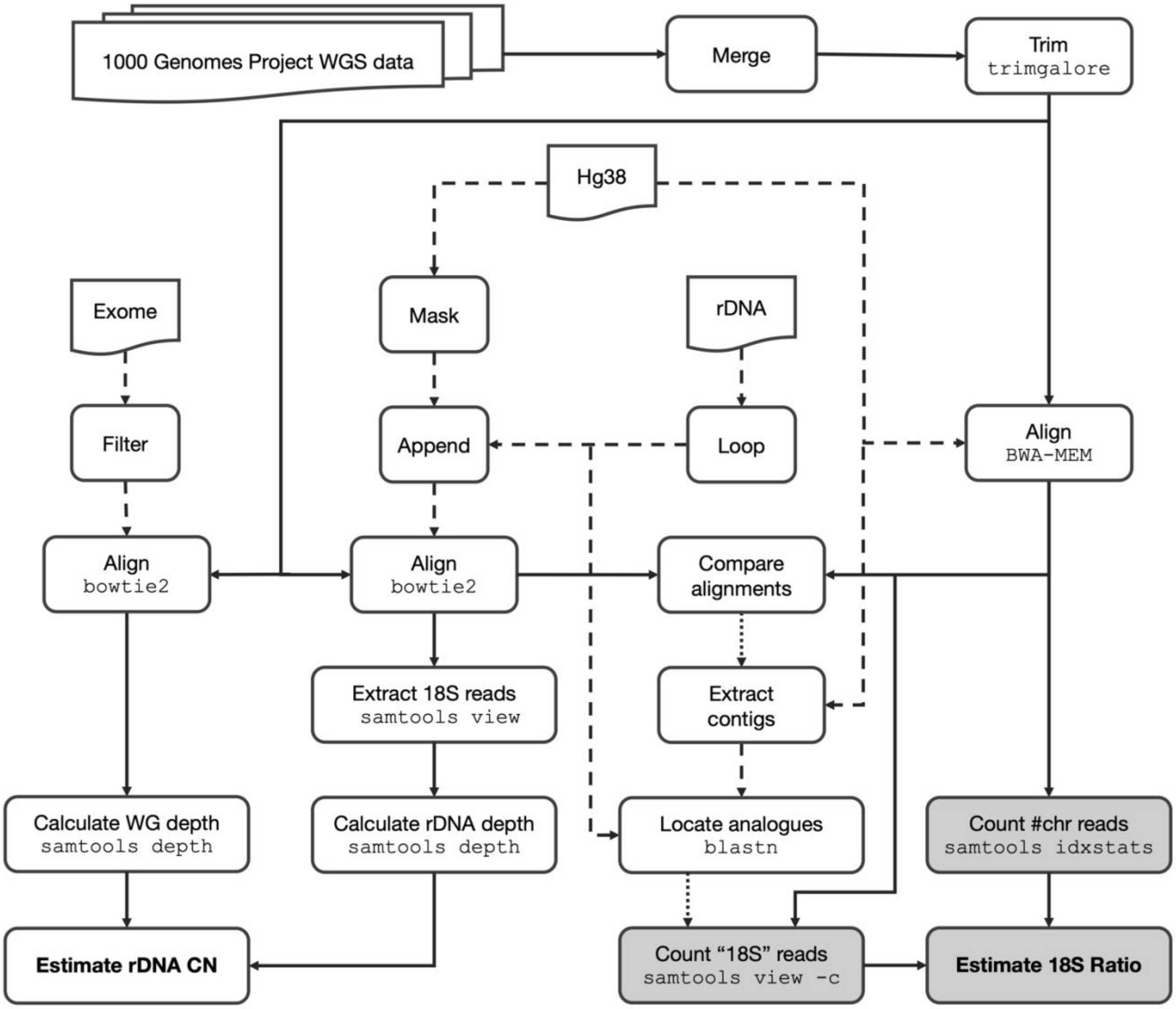

Figure S1. Schematic representation of the methodology employed to derive rDNA CN proxy estimates; Related to Figure 1 and STAR Methods. Solid lines indicate the flow of sequencing data, dashed lines the flow of reference sequences, and dotted lines the flow of coordinates. Boxes with grey background represent steps conducted on UK Biobank data to derive their corresponding 18S Ratios from the available `BWA-MEM` alignments.

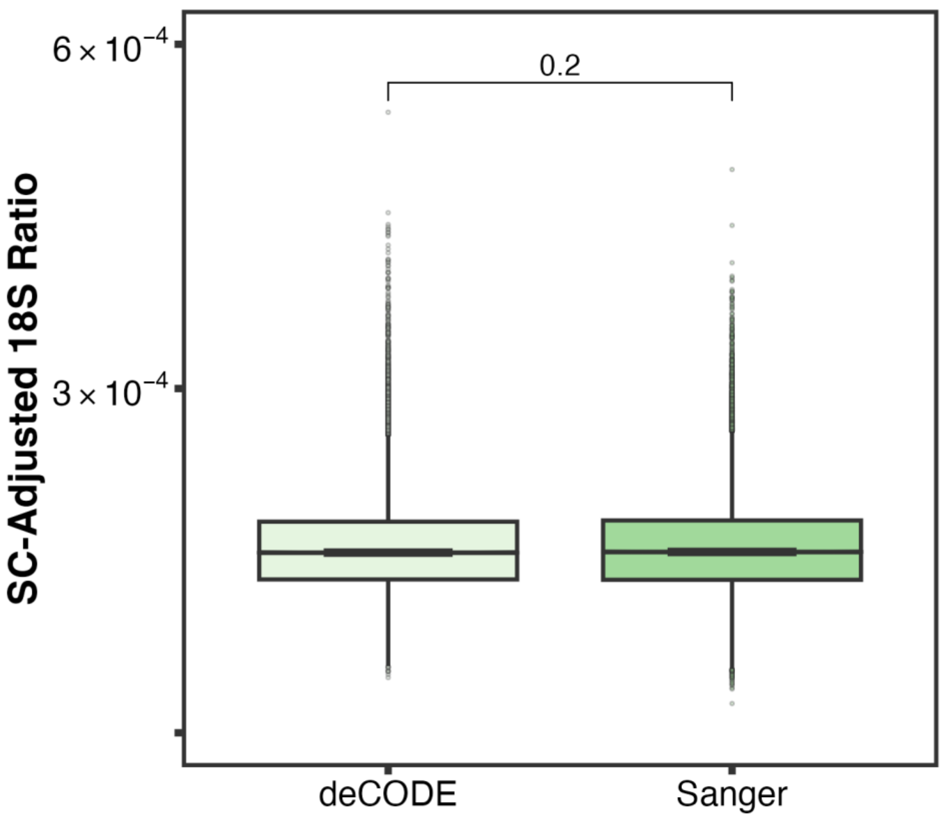

Figure S2. Comparison between sequencing centre-adjusted 18S Ratios; Related to Figure 1 and STAR Methods. No significant difference in mean 18S Ratio (Wilcoxon signed-rank test, $p = 0.2$) between UKB participants from the first WGS release sequenced by deCODE Genetics or at the Sanger Institute after adjusting the values accordingly.

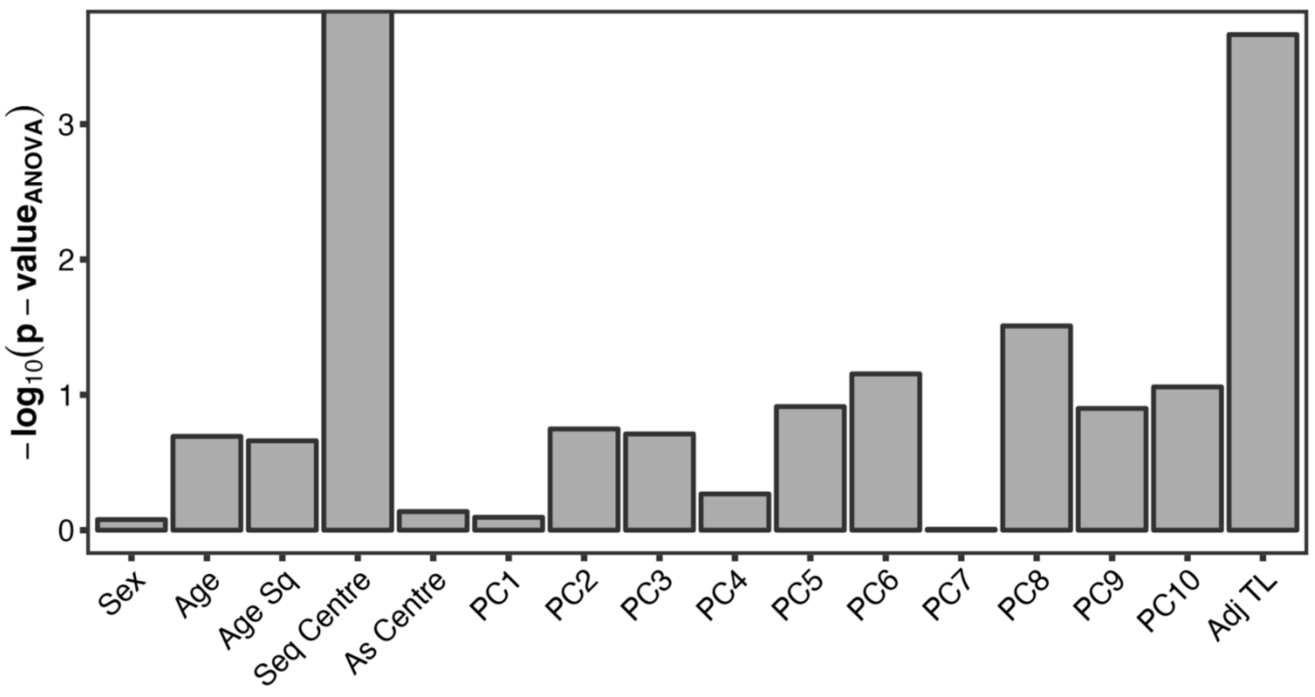

Figure S3. ANOVA p-values for association with 18S Ratio in WB individuals from the first WGS release; Related to Figure 1. Variables considered here are sex, age, age squared ("Age Sq"), sequencing centre ("Seq Centre"), assessment centre ("As Centre"), the first 10 genetic principal components ("PC1" to "PC10"), and adjusted telomere length ("Adj TL"). Significance level for the sequencing centre factor is below the minimum threshold that `R` can report ($p < 10^{-300}$), and thus its bar is represented cropped.

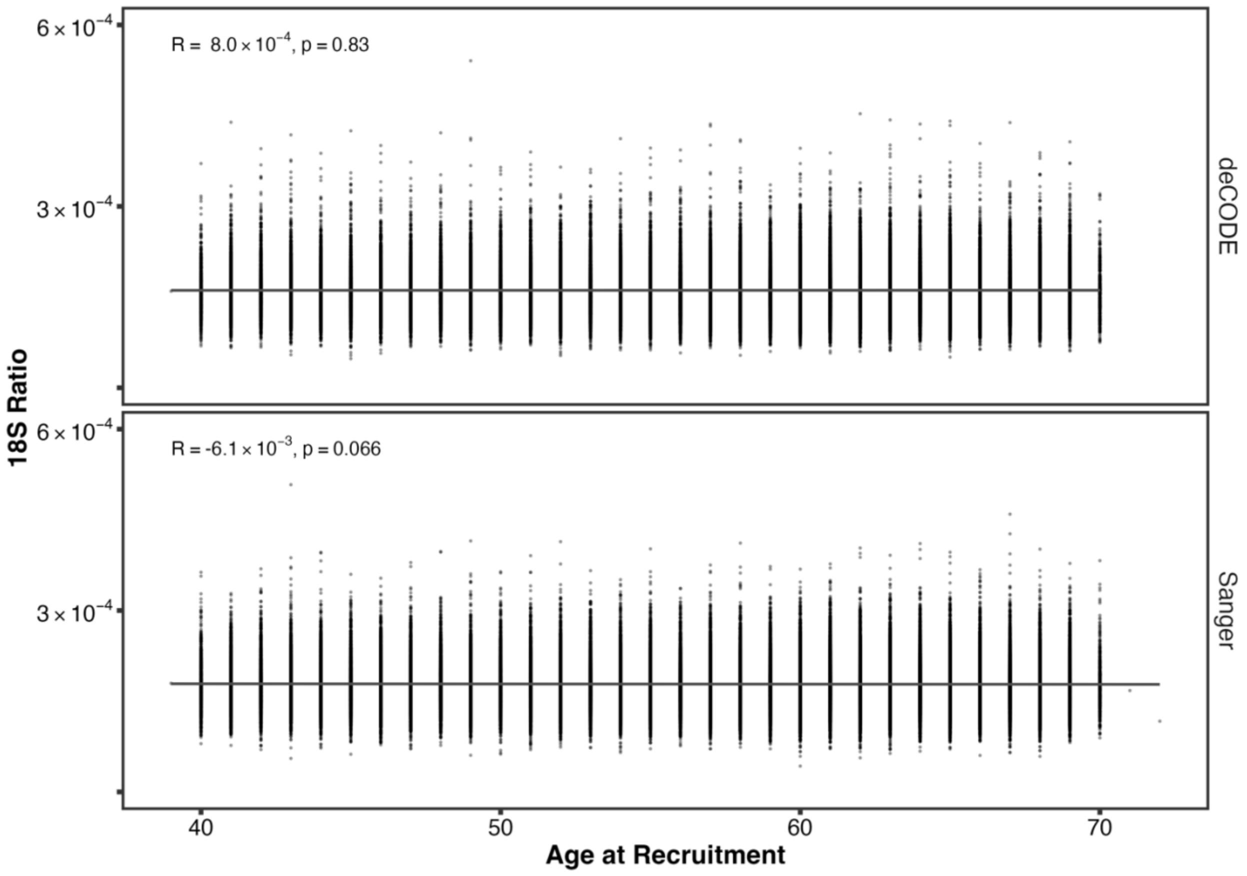

Figure S4. Correlation between 18S Ratio and age in WB individuals; Related to Figure 1. No statistically-significant Pearson's correlation between 18S Ratio and age at recruitment in white British UK Biobank participants from the first WGS release in either sequencing centre.

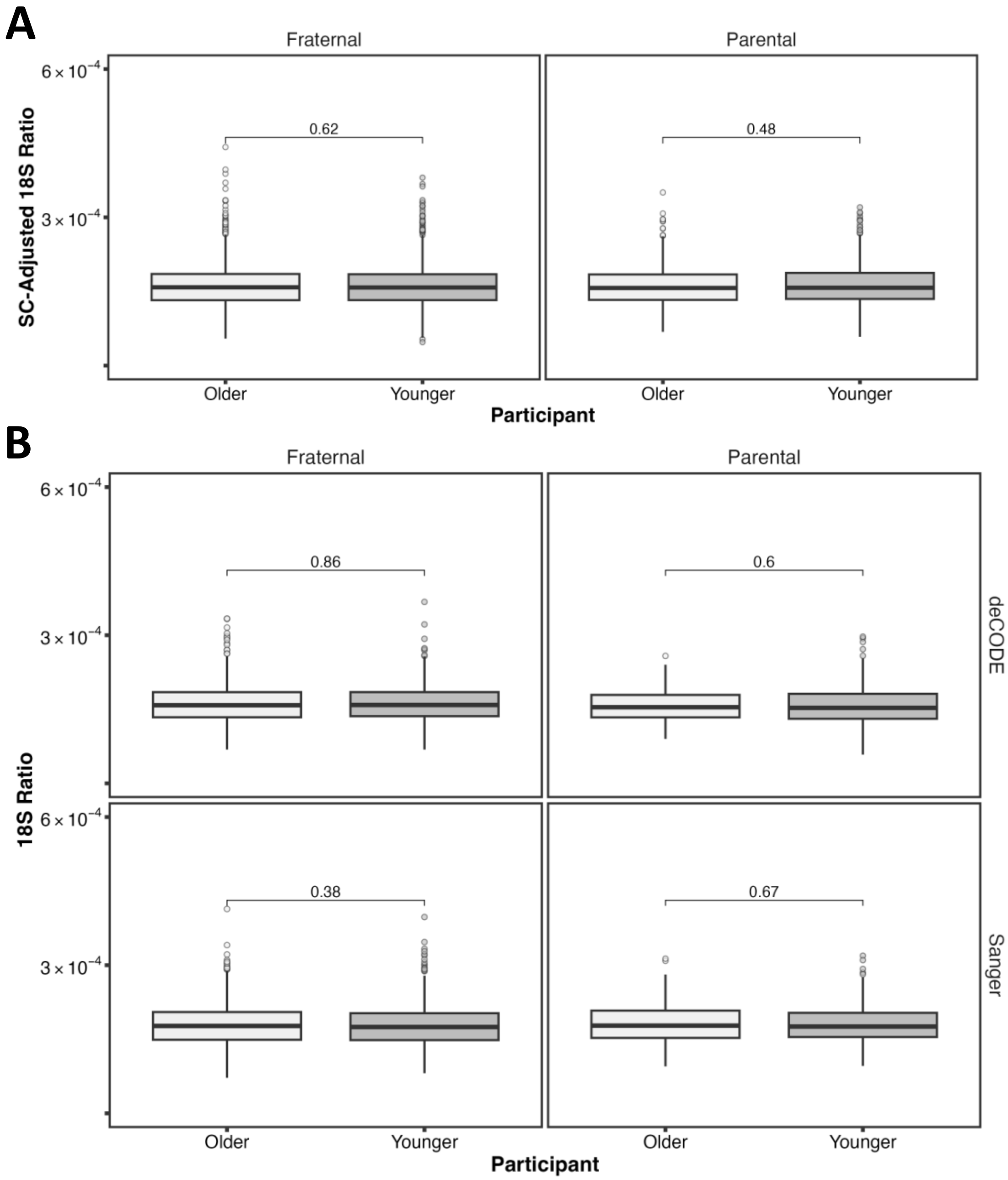

Figure S5. Comparison between 18S Ratios in first-degree relative pairs; Related to Figure 1. No significant difference (paired Wilcoxon signed-rank test) in **(A)** mean sequencing centre-adjusted 18S Ratio between younger and older first-degree relatives for each pair of sequenced WB individuals in the first UKB WGS release and **(B)** mean unadjusted 18S Ratio in pairs where both individuals were sequenced by the same centre, split by type of relationship: fraternal (N = 3,270 overall pairs, 1,043 in Sanger, 694 in deCODE) or parental (N = 905 overall pairs, 297 in Sanger, 218 in deCODE).

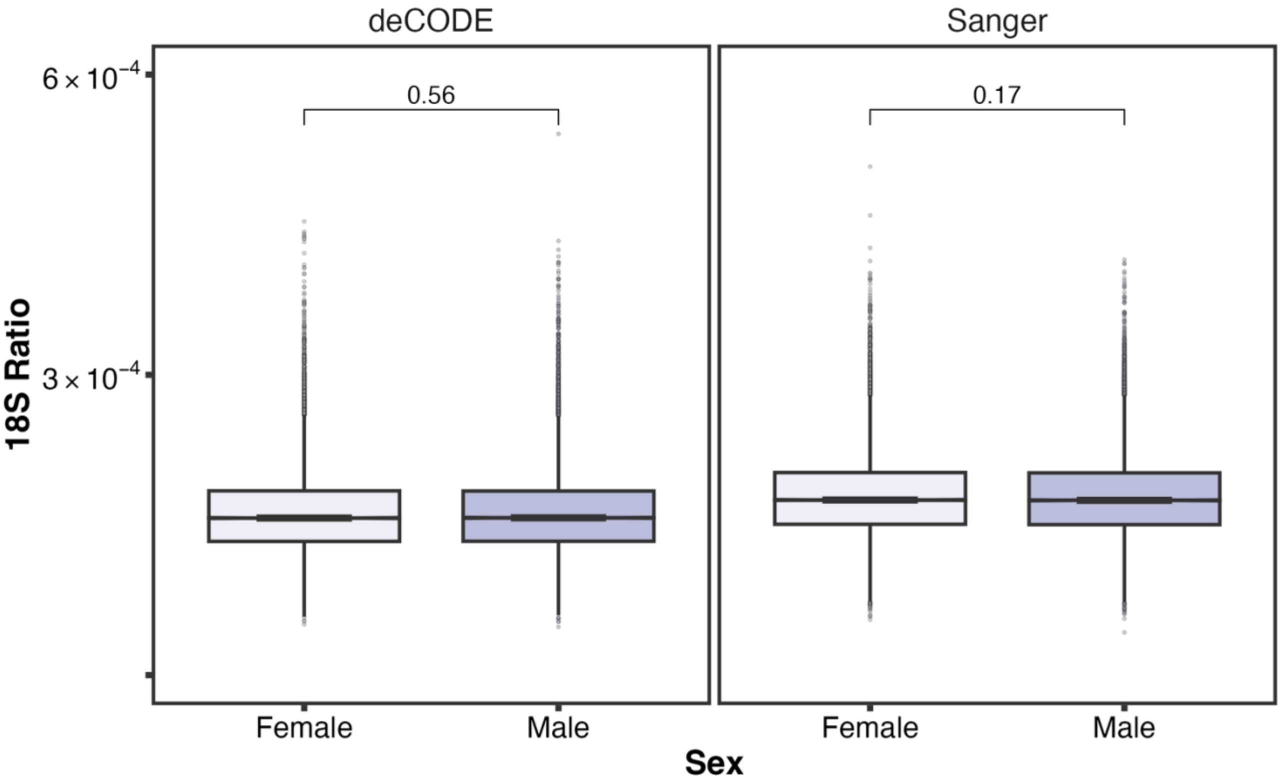

Figure S6. Comparison of 18S Ratios across sexes; Related to Figure 1. No statistically-significant difference in mean 18S Ratio distributions across sexes in white British UKB participants from the first WGS release in either sequencing centre.

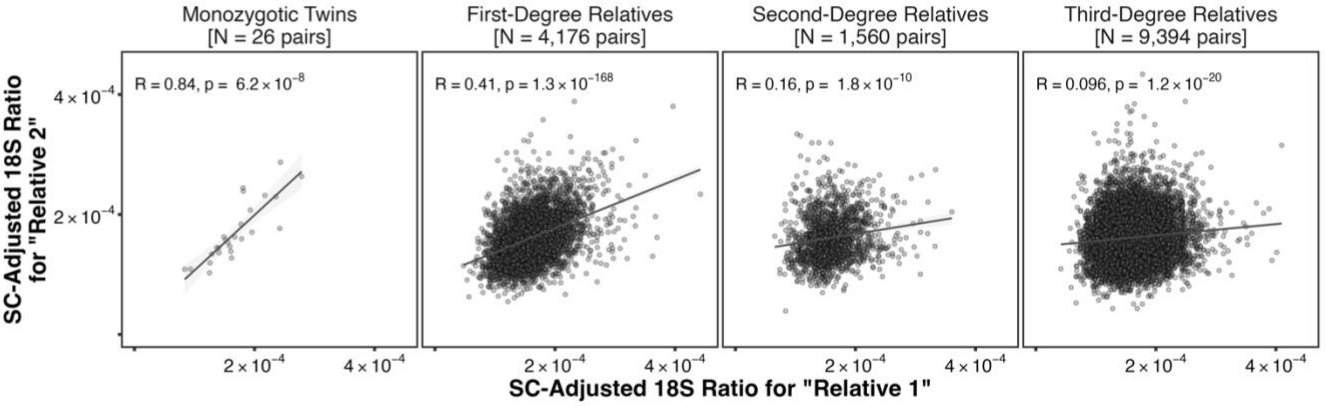

Figure S7. Comparison between sequencing-centre adjusted 18S Ratios in relatives; Related to Figure 1. Pearson's correlation calculated on pairs of genetically identified white British UKB relative pairs from the first WGS release, with 18S Ratios adjusted for sequencing centre effects as in **fig. S2**.

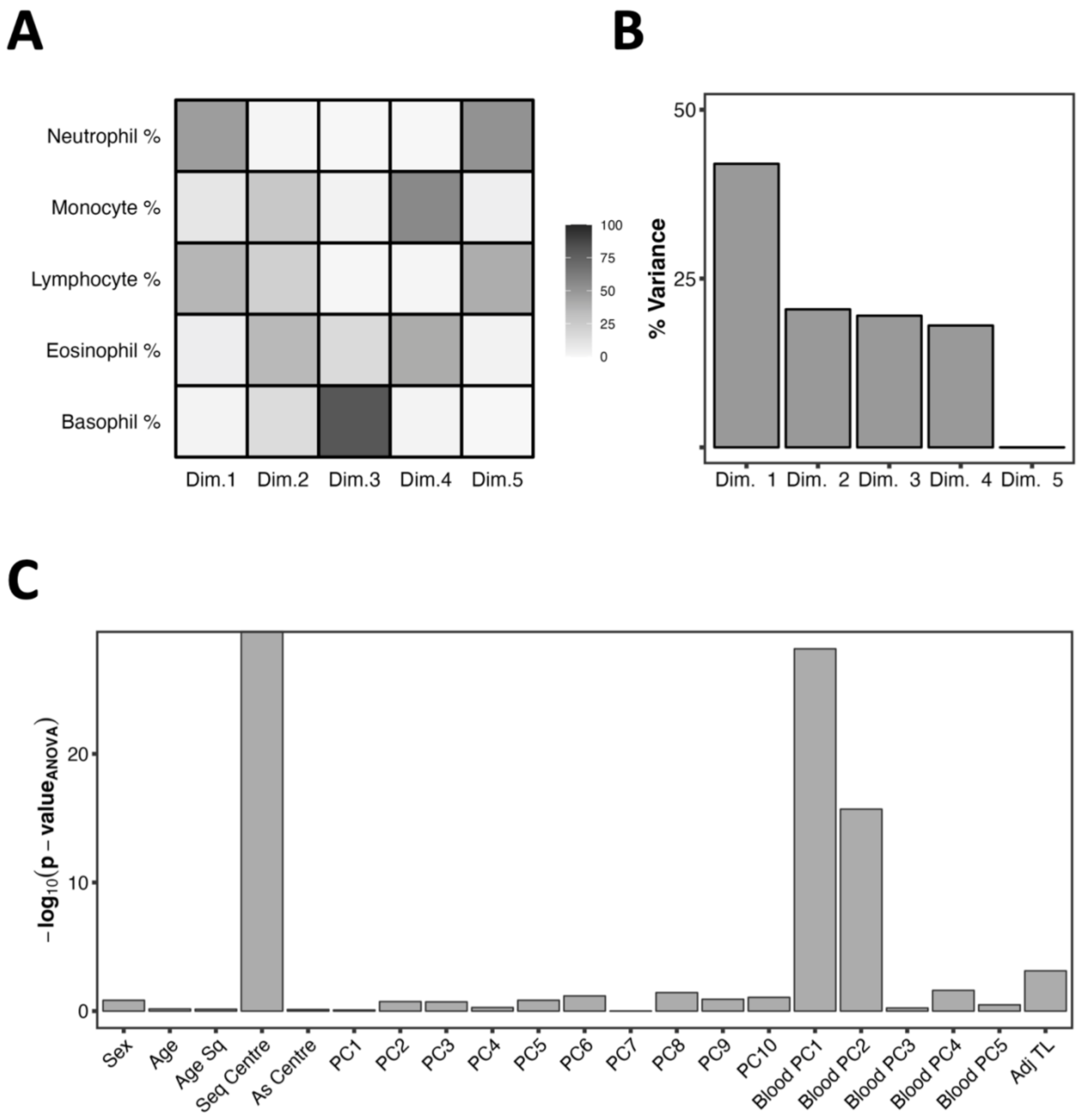

Figure S8. Characterisation of the blood composition principal components; Related to Figure 1. **(A)** Contribution of the percentage of each nucleated blood cell subtype to each of the five principal component dimensions. **(B)** Percentage of variance in the population explained by each of the five principal components obtained from blood cell subtype proportions. **(C)** ANOVA p-values for association with 18S Ratio in WB individuals from the first WGS release, including principal components obtained from blood cell subtype proportions (“Blood PC1” to “Blood PC5”). Interpretation as in **fig. S3**.

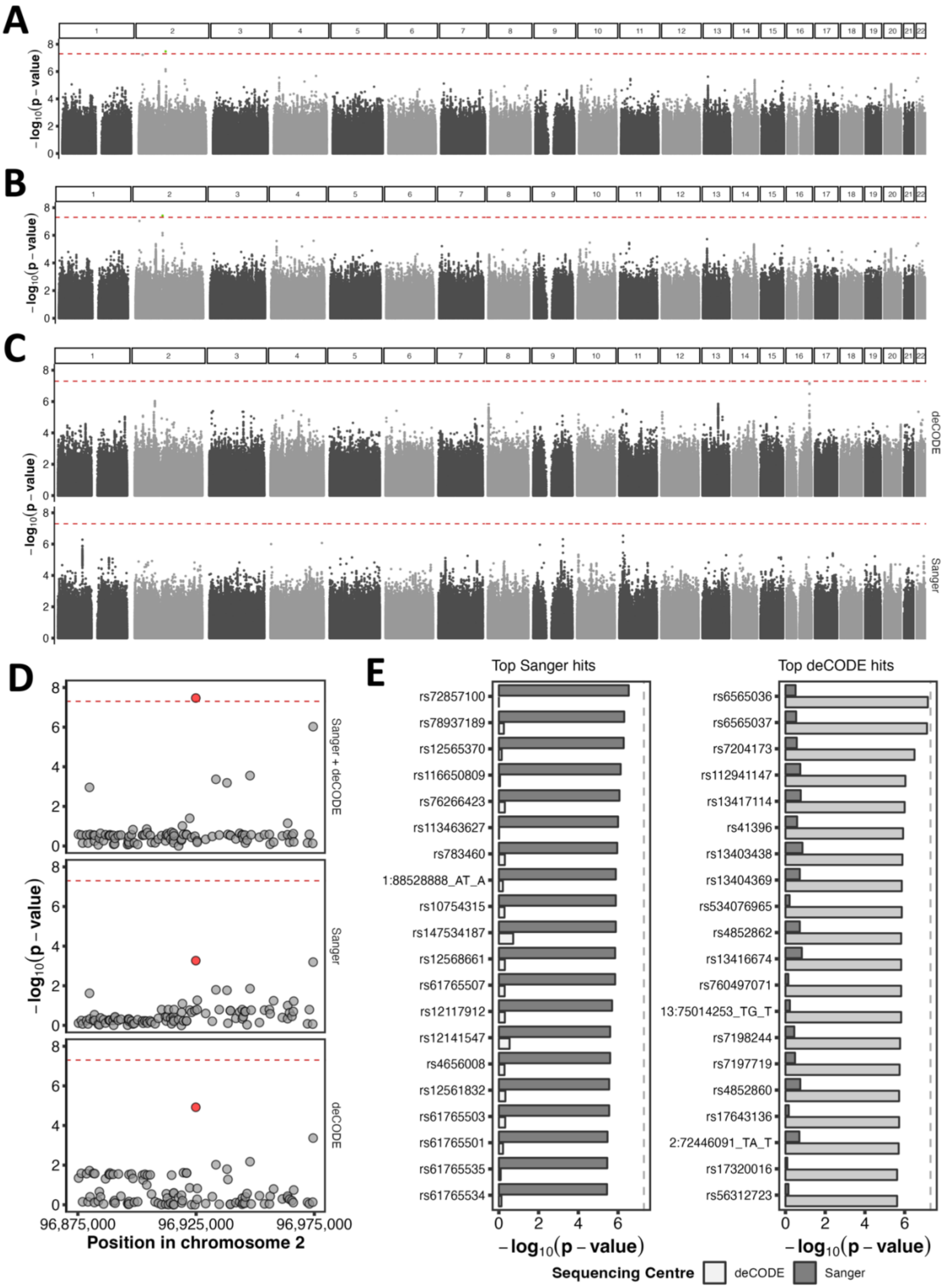

Figure S9. Expanded GWAS results; Related to Figure 1. (Full caption on next page)

Figure S9. Expanded GWAS results; Related to Figure 1. Manhattan plot for GWAS of 18S Ratio in WB participants from the first WGS release **(A)** with and **(B)** without blood principal components as covariates, and **(C)** with blood principal components as covariates but split by sequencing centre. Interpretation as in **Fig. 1G**. **(D)** Zoom in of GWAS results in the region 50kb upstream and downstream of rs62153030 in chromosome 2, indicated with a red dot. The expected gradual increase and decrease in significance due to co-inheritance at both sides of the significant locus is not observed regardless of whether the analysis is conducted on all WB participants or split by sequencing centre. **(E)** Significance level in the sequencing-centre specific analyses of (B) for the 20 most significant loci in "Sanger" (Left) and "deCODE" (Right) individuals. No variant is common between the two lists, and s62153030 does not appear in either.

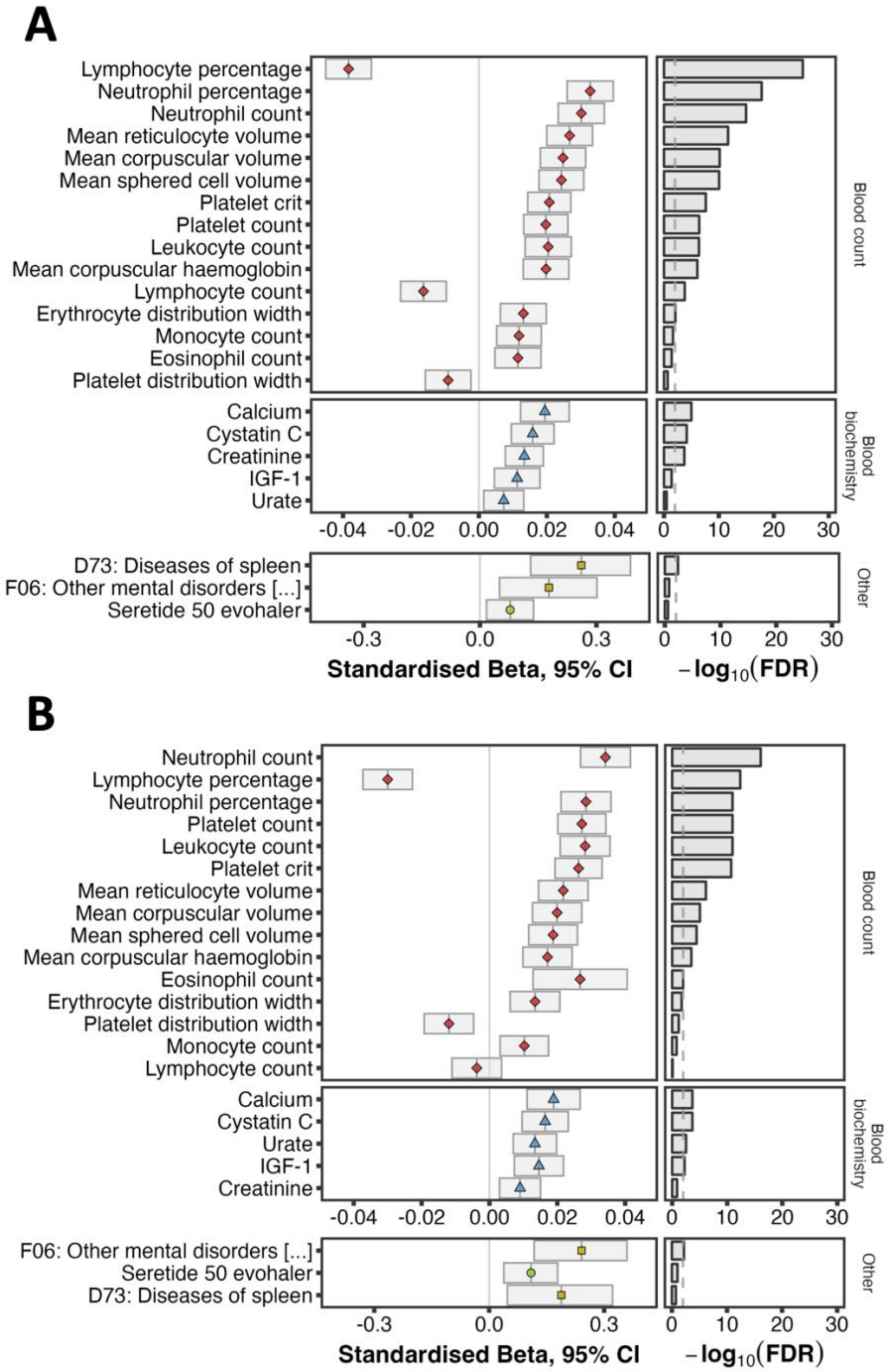

Figure S10. Sequencing centre-specific results for FDR-significant hits in phenome-wide screen of 18S Ratio associations; Related to Figure 2. For subsets of white British individuals from the first UKB WGS release with sequencing data generated either by **(A)** Sanger or **(B)** deCODE, effect size (Left) and significance level adjusted for multiple testing (Right) reported by PHESANT for the association between 18S Ratio and the phenotypes reaching FDR-significance in the overall analysis of **Fig. 2A.**

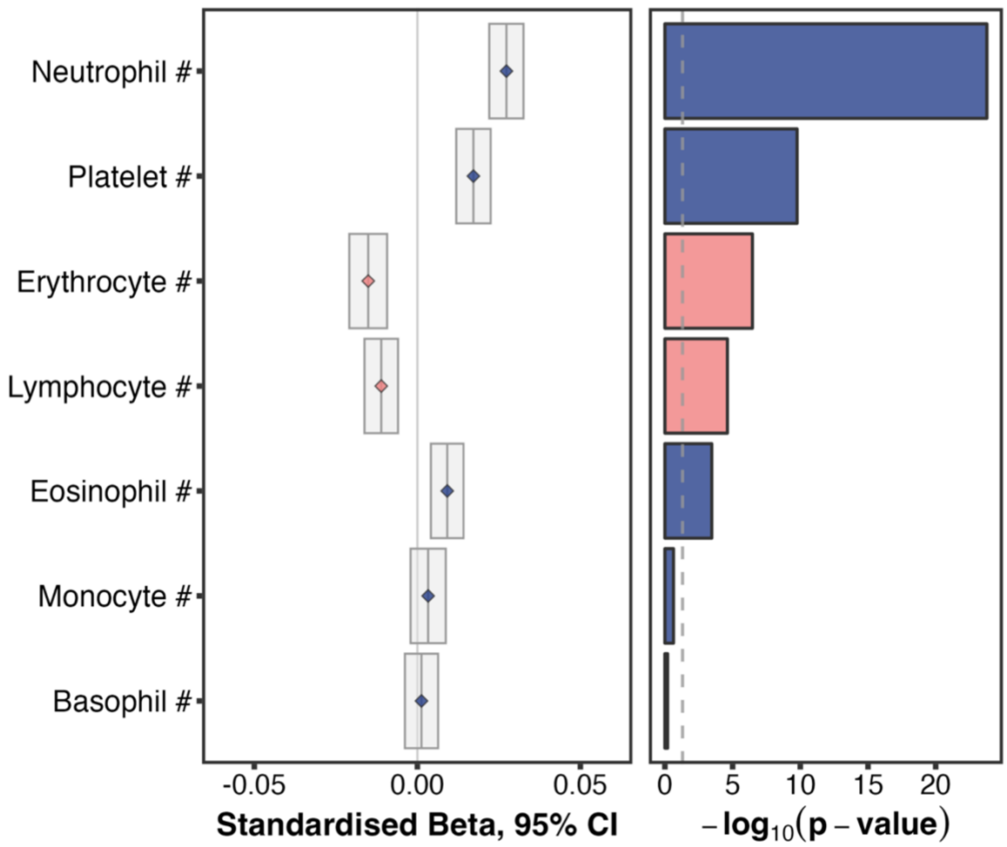

Figure S11. Multivariate association between blood cell subtype counts and 18S Ratio; Related to Figure 2. Effect size and corresponding 95% confidence intervals (Left) and significance level (Right) for the association between blood cell subtype counts and 18S Ratio on white British individuals from the first UKB WGS release, calculated on a linear model including 18S Ratio as response and all these cell counts as explanatory variables alongside covariates.

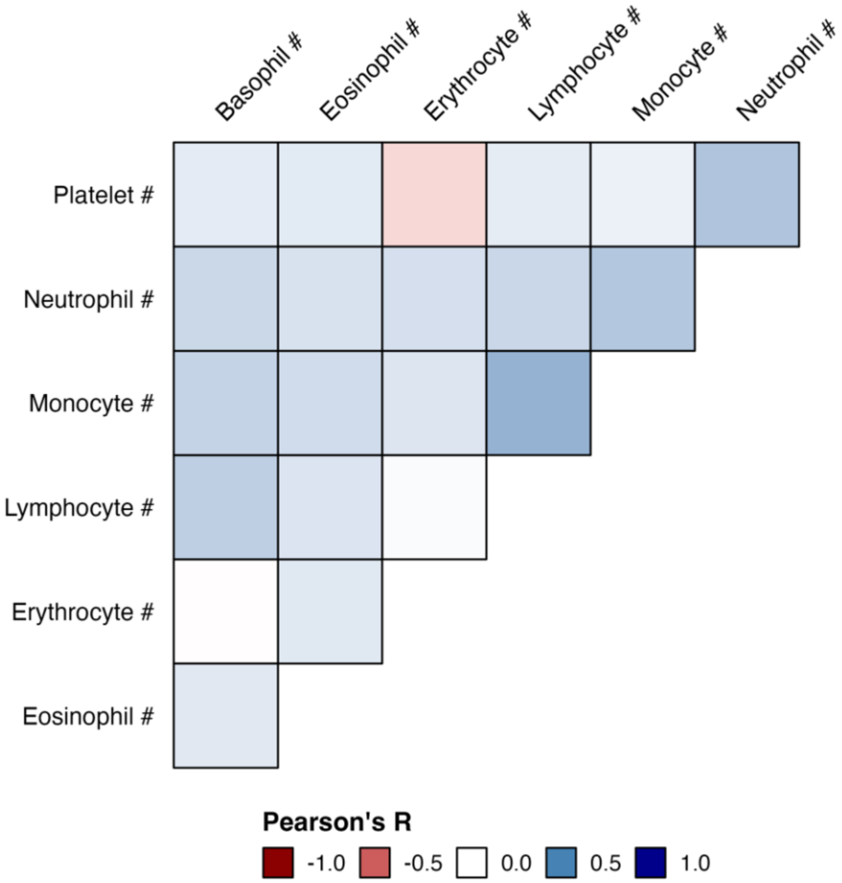

Figure S12. Correlation levels between blood cell subtype counts in the UK Biobank; Related to Figure 2. Pearson's R obtained from pairwise correlations between cell counts on N = 477,163 UKB participants. All corresponding significance levels are below 10^{-295} except for erythrocytes versus platelets ($p = 5.46\times10^{-26}$) and basophils ($p = 1.28\times10^{-5}$).

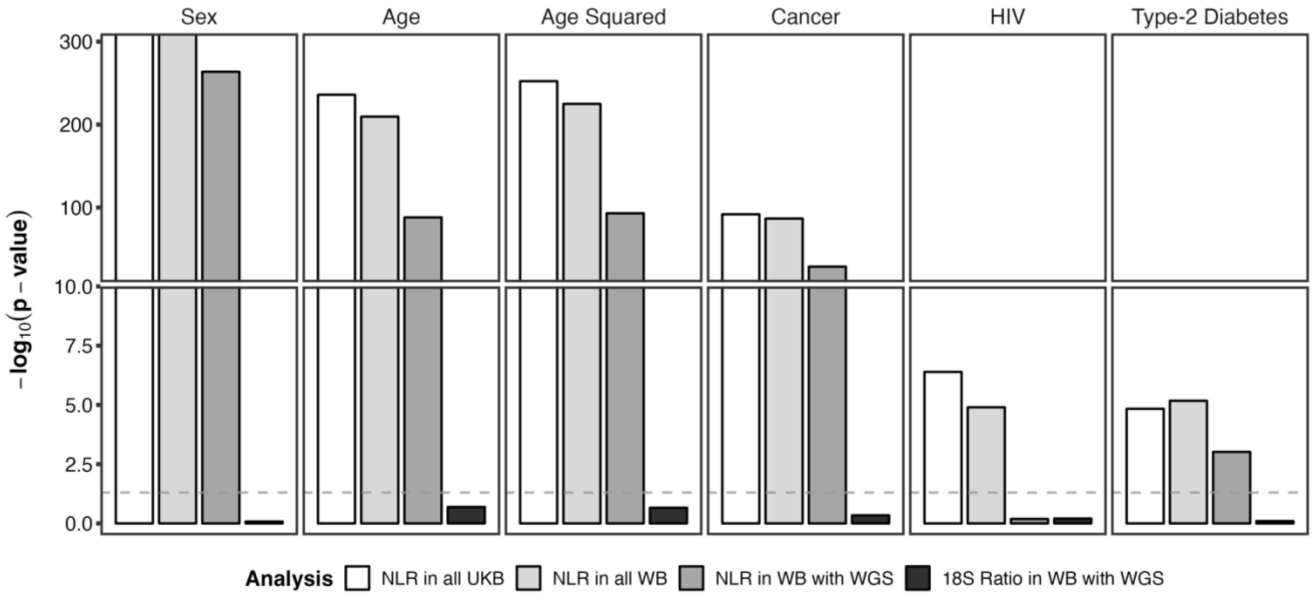

Figure S13. Association with drivers of blood cell composition changes; Related to Figure 2. Extended version of **Fig. 2D**, including a panel for Age Squared as further driver and white bars displaying the significance levels in associations using all UKB participants.

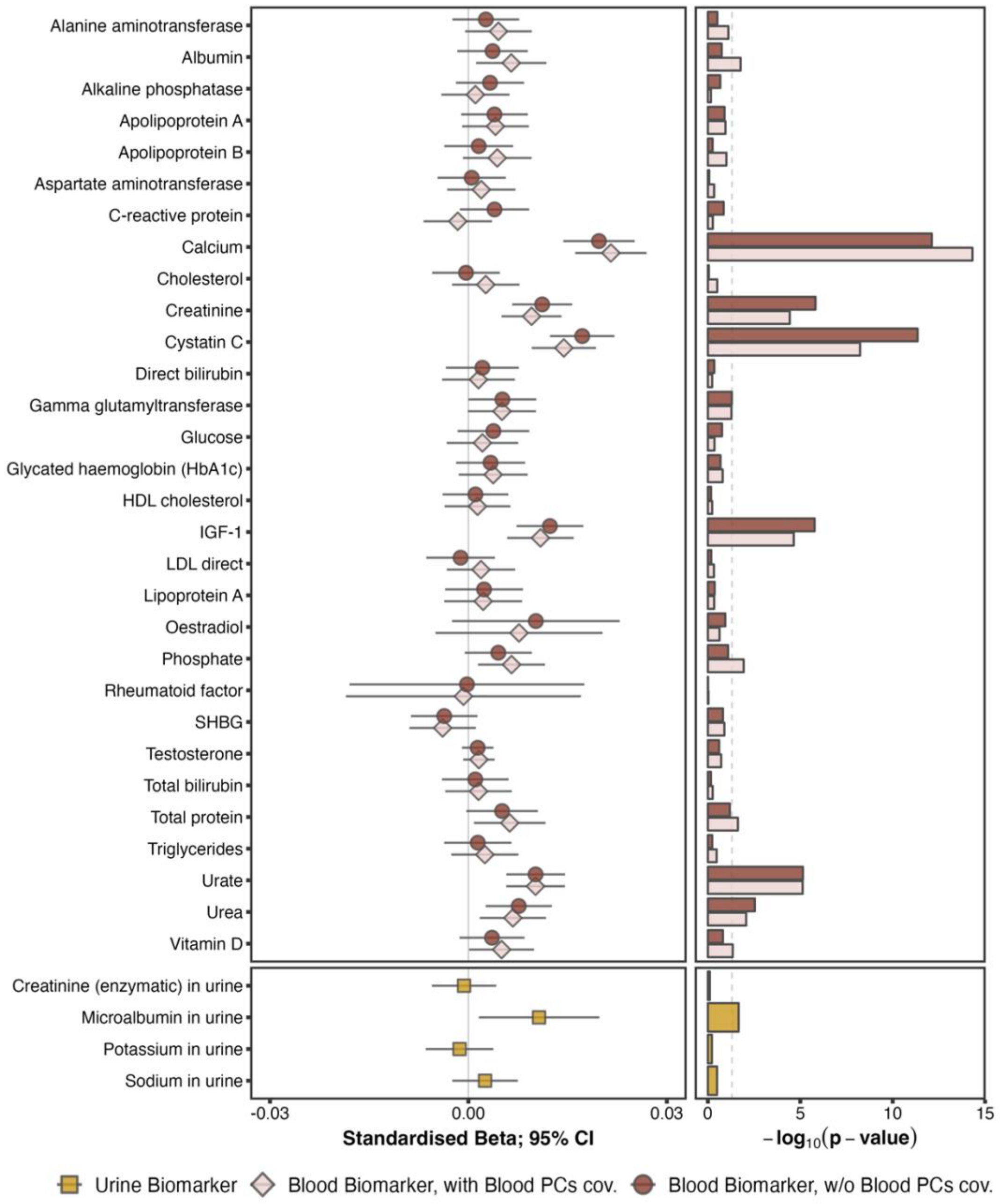

Figure S14. Association between 18S Ratio and biochemistry biomarker measurements; Related to Figure 4. Effect size and corresponding 95% confidence intervals (Left), and significance level (Right) for the association between 18S Ratio and each blood and urine biochemistry biomarker in the UKB obtained from linear models with targeted covariates fit on white British participants from the first WGS release, regardless of their recorded statin intake. Interpretation as in **Fig. 4A**.

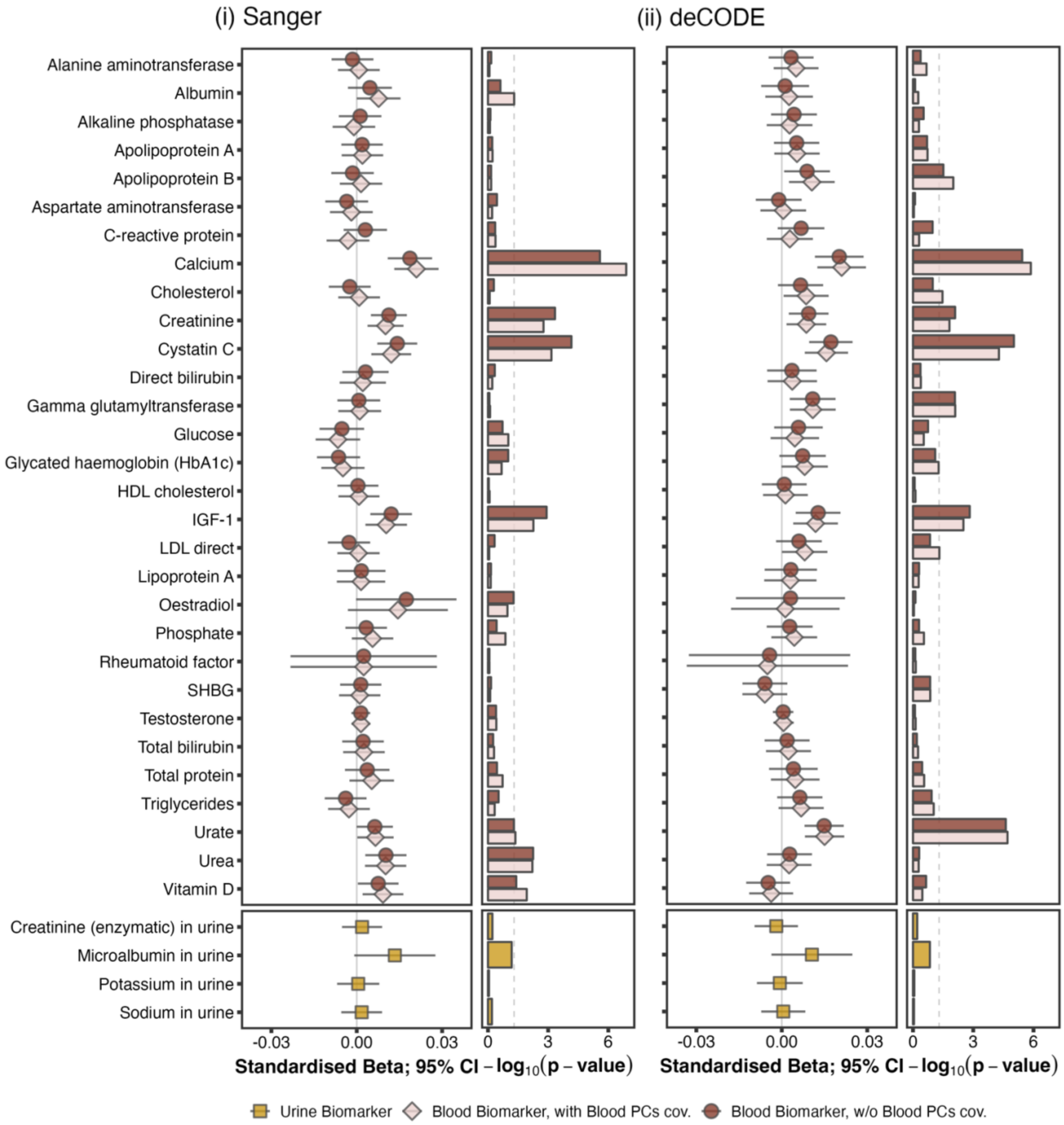

Figure S15. Association between 18S Ratio and biochemistry biomarker measurements on participants not taking statins, split by sequencing centre; Related to Figure 4. For subsets of white British individuals from the first UKB WGS release not recorded as taking statins and with sequencing data generated either by **(i)** Sanger or **(ii)** deCODE, effect size and corresponding 95% confidence intervals (Left), and significance level (Right) for the association between 18S Ratio and each blood and urine biochemistry biomarker in the UKB. Interpretation as in **Fig. 4A**.

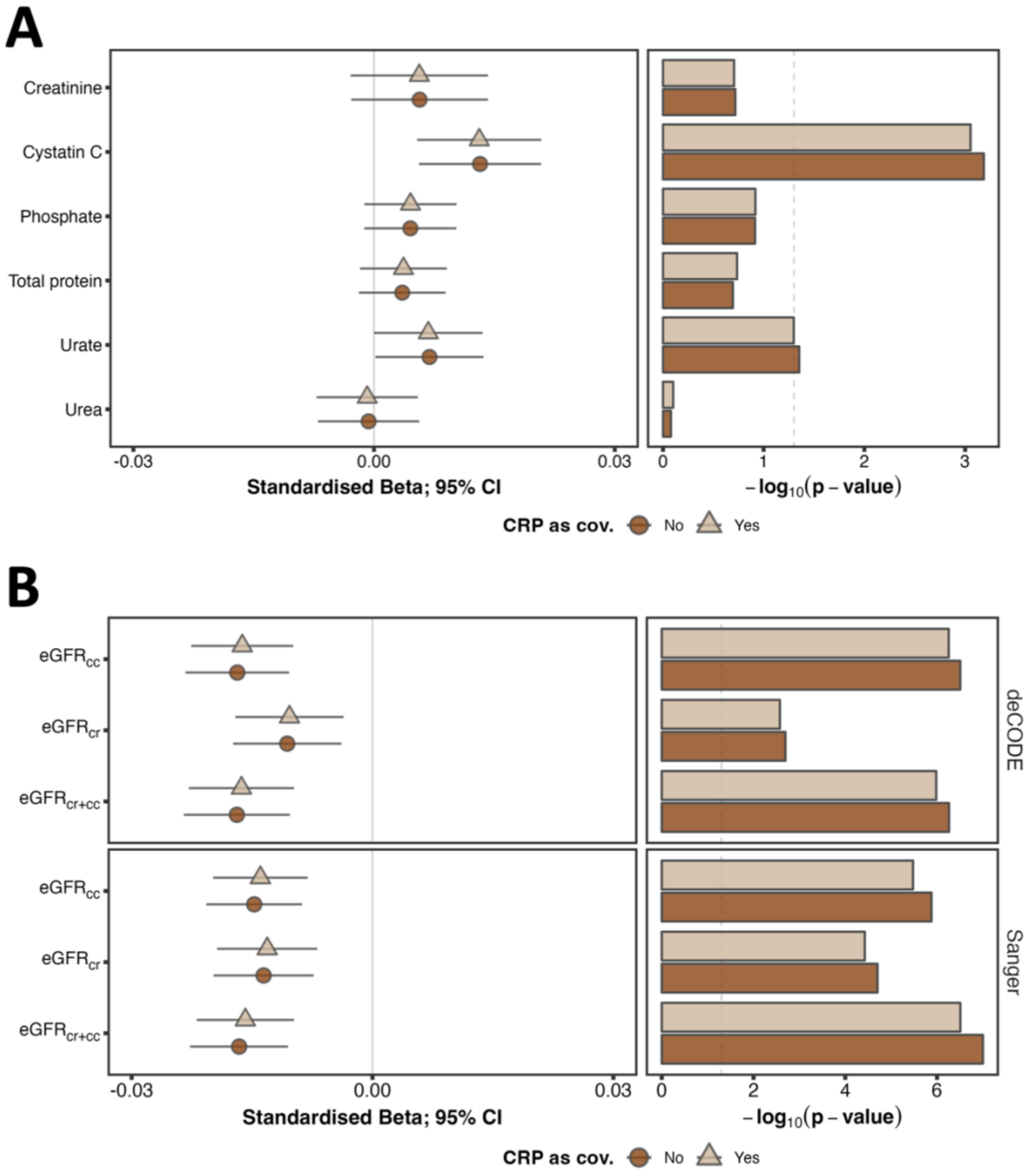

Figure S16. Association between 18S Ratio and kidney function markers; Related to Figure 4. Effect size and corresponding 95% confidence interval (Left) and significance level (Right) for the association in white British UKB individuals from the first WGS release between 18S Ratio and **(A)** kidney function-related blood biomarkers in a multivariate linear model with 18S Ratio as response variable, and (B) Glomerular Filtration Rate estimates, split by sequencing centre. Interpretation as in **Fig. 4B**.

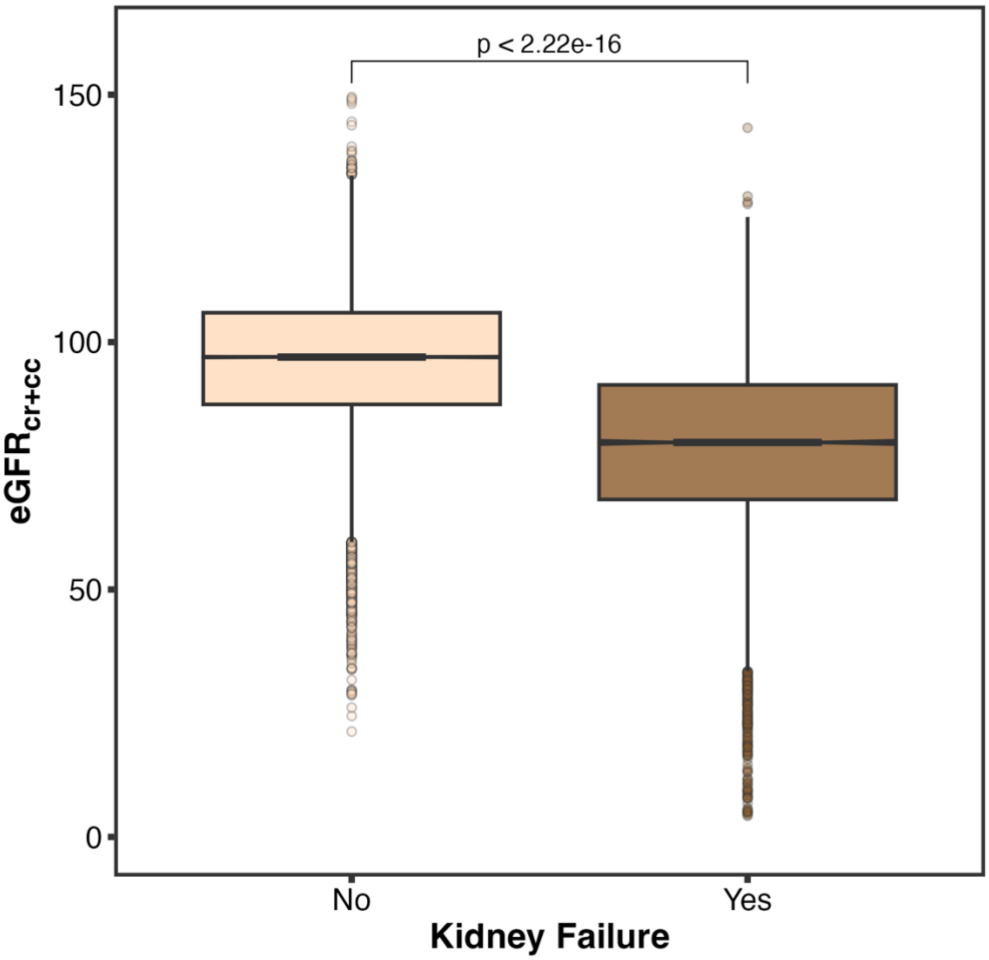

Figure S17. Comparison of Glomerular Filtration Rate estimates in UKB participants with and without reported Kidney Failure; Related to Figure 4. eGFR values obtained from both Creatinine and Cystatin C measurements are significantly lower in participants with acute (N17) and/or chronic (N18) kidney failure registered in the UKB (N_{Yes} = 12,093; N_{No} = 138,285; Wilcoxon signed-rank test, $p < 10^{-300}$).

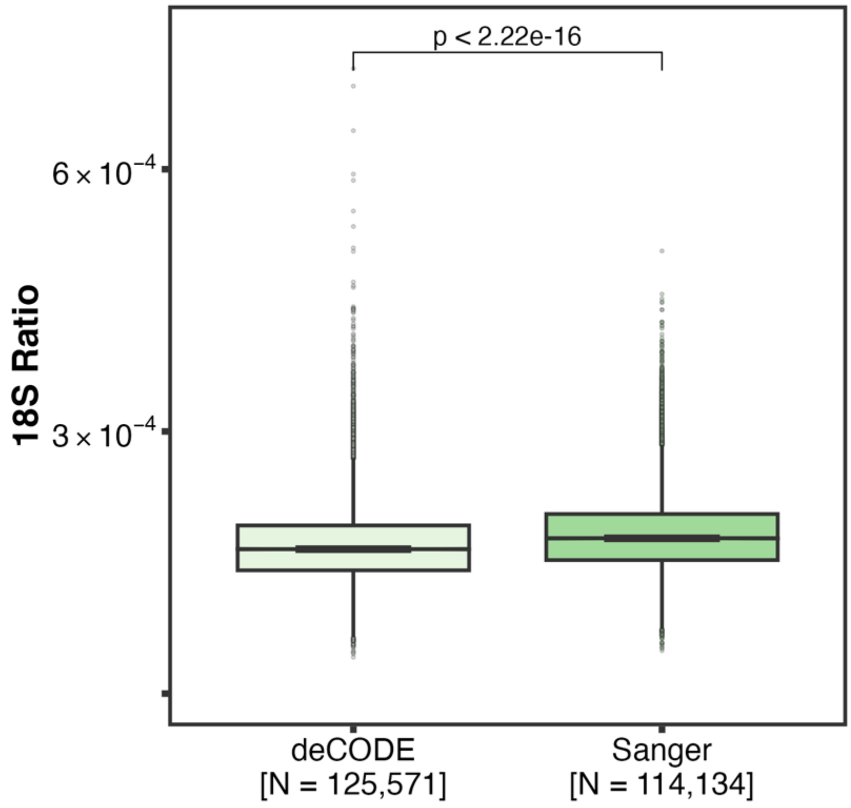

Figure S18. Comparison of 18S Ratio estimates across sequencing centres in the second UKB WGS release; Related to Figure 5. Statistically-significant differences in 18S Ratios remain between UKB participants sequenced by deCODE Genetics or at the Sanger Institute in the second UKB WGS release (N_{deCODE} = 125,571; N_{Sanger} = 114,124; Wilcoxon signed-rank test, $p < 10^{-300}$).

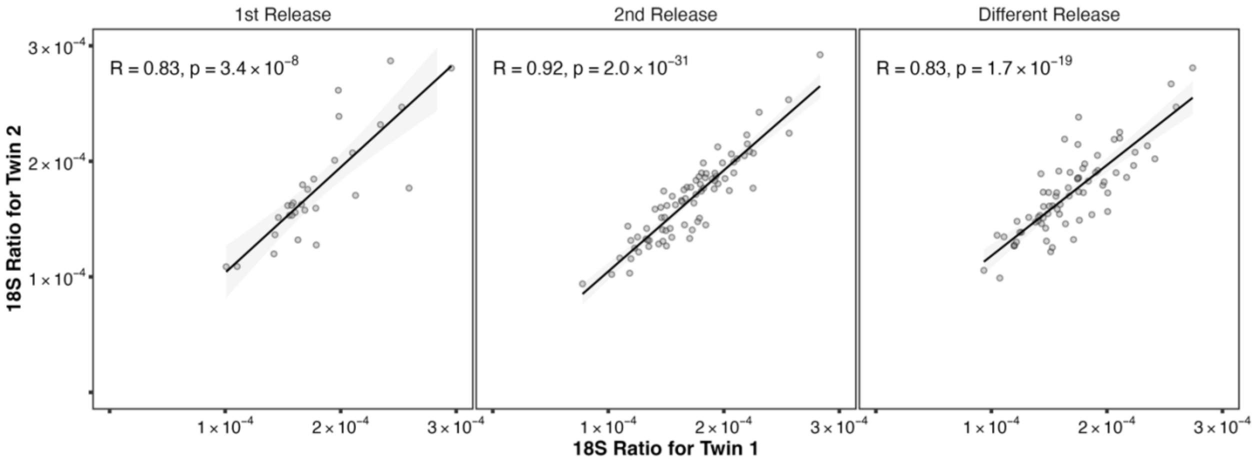

Figure S19. Comparison of twin 18S Ratio estimates within and across UKB WGS releases; Related to Figure 5. For pairs of genetically-identified twins from the UKB, Pearson's correlation calculated on pairs with both individuals appear on either the first (Left) or the second (Middle) WGS release, or where each appears on a different release (Right).

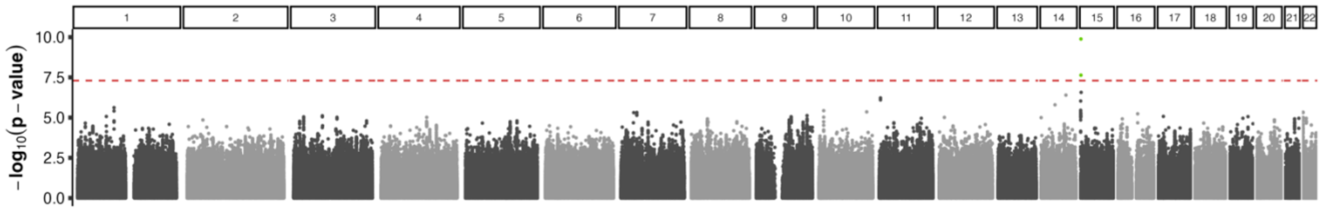

Figure S20. GWAS results in the second UKB WGS release; Related to Figure 5. Manhattan plot for GWAS of 18S Ratio in unrelated white British participants in the second UKB WGS release. Interpretation as in **Fig. 1G**.

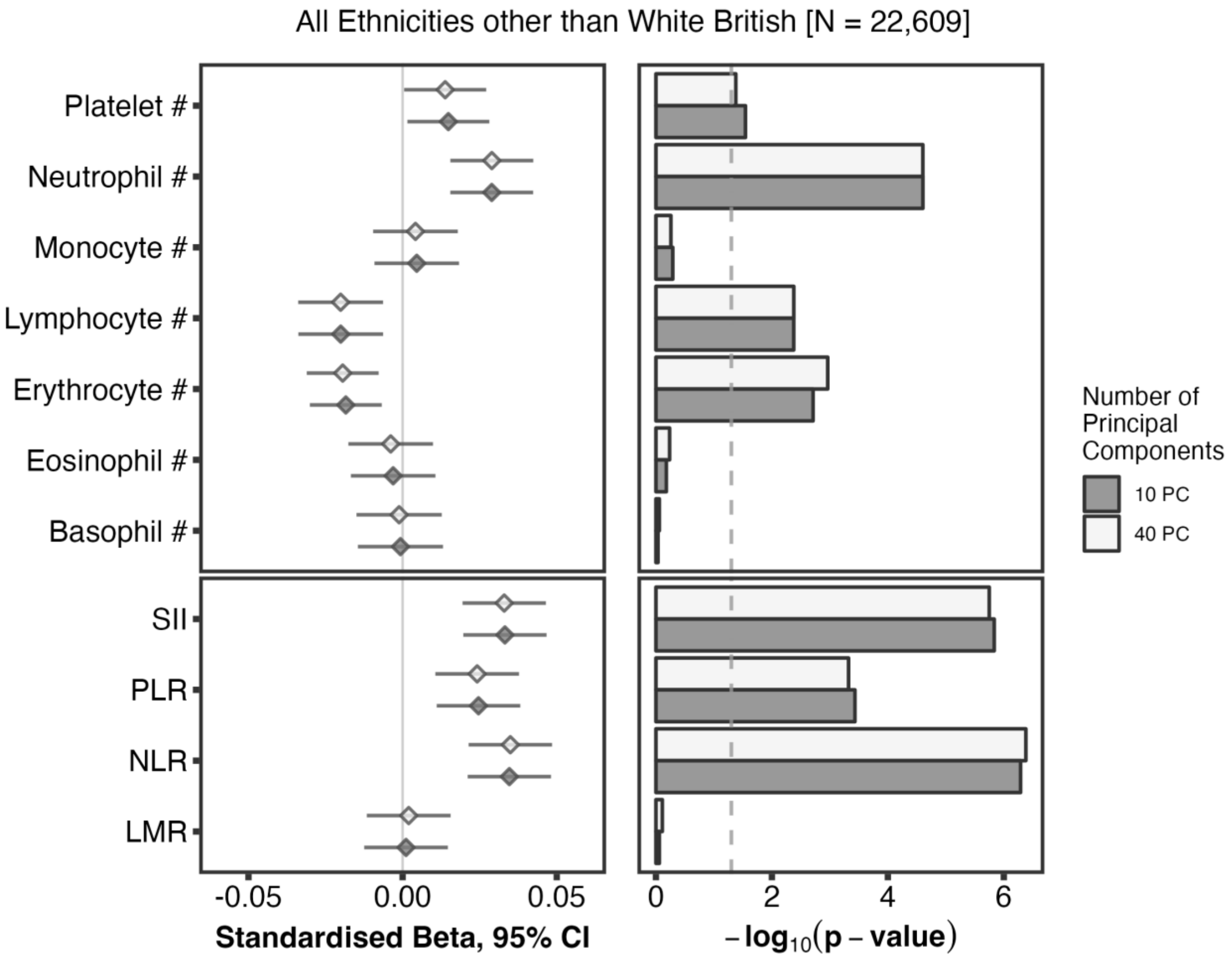

Figure S21. Effect of the number of genetic principal components on the association between 18S Ratio and blood composition; Related to STAR Methods. Effect size (Left) and significance levels (Right) for associations with 18S Ratio in N = 22,609 UKB participants of non-WB self-reported ethnicities from the first WGS release obtained on linear models with the first 10 (dark grey; same values as in **Fig. 3E**) or all 40 available genetic principal components (light grey) included as covariates.

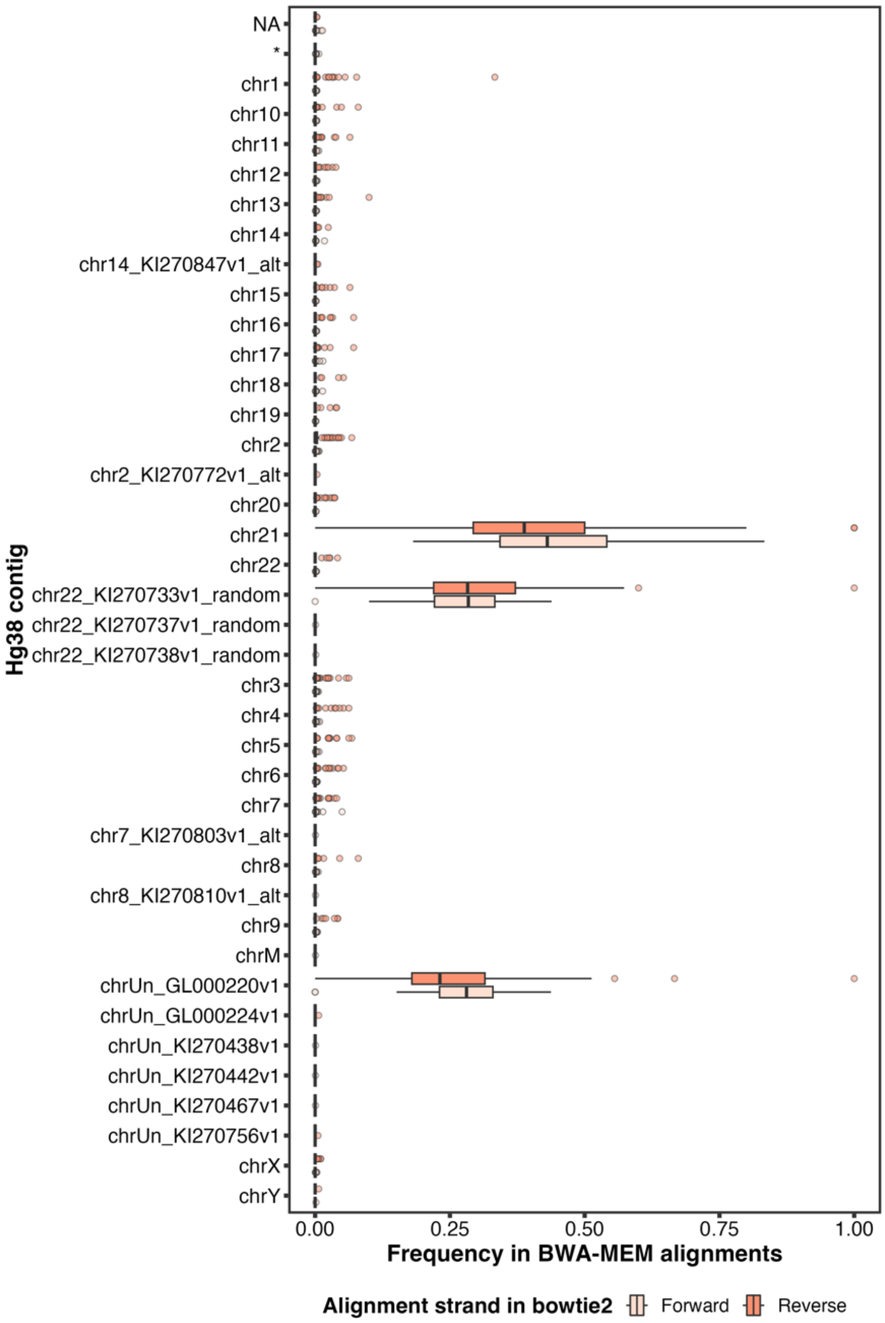

Figure S22. Identification of Hg38 contigs harbouring rDNA analogues; Related to STAR Methods. For each of 94 GBR samples from the 1000 Genomes Project, proportion of forward (light orange) and reverse (dark orange) strand reads overlapping position 7980 of the KY962518.1 human rDNA reference when aligned to a tailored assembly with `bowtie2` mapping at each contig when aligned to the Hg38 assembly using `BWA-MEM`. "NA" indicates reads that did not appear on the `BWA-MEM` output, whereas "*" represents reads whose "mapping position" is "not available", according to the `samtools` documentation.

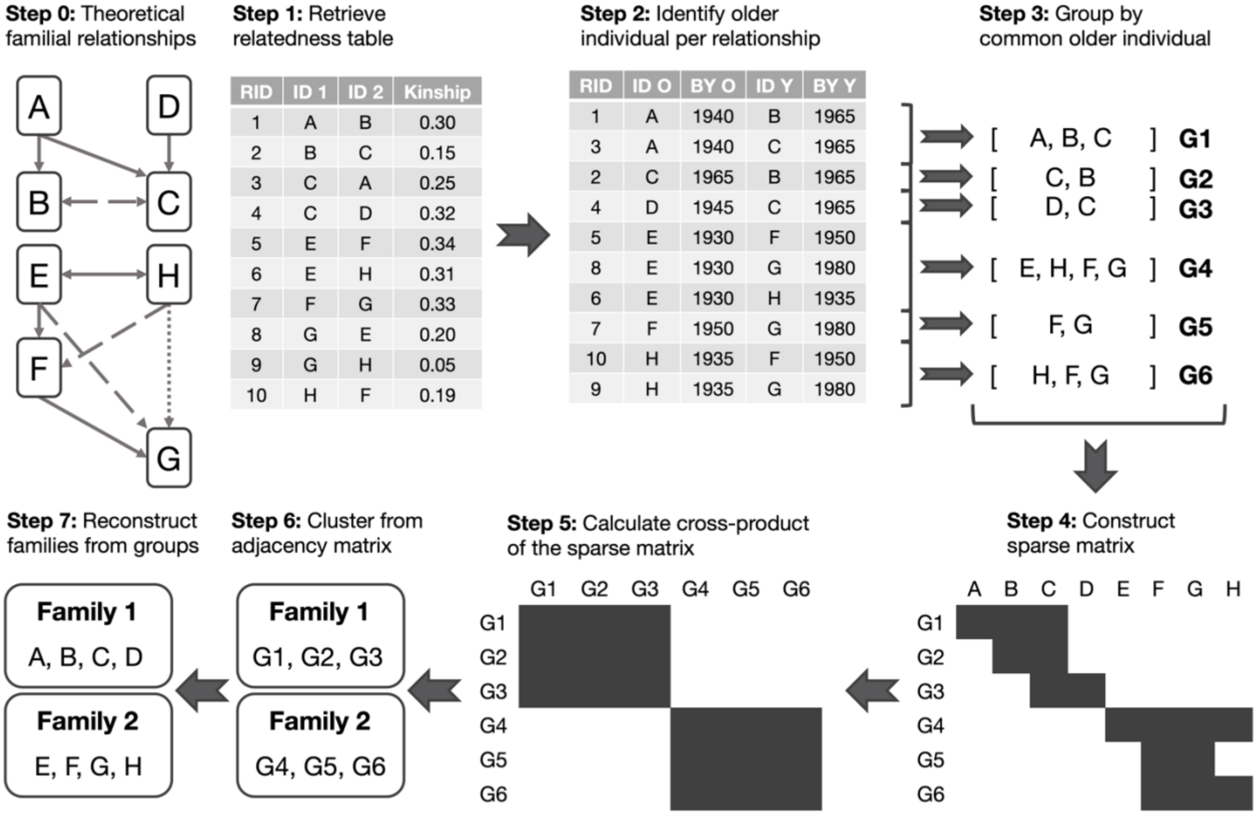

Figure S23. Hypothetical example of the procedure followed to group UKB participants into families; Related to STAR Methods. Given two hypothetical family groups with relationships of different degrees (Step 0; solid line for first degree, dashed for second degree, and dotted for third degree) and their corresponding entries from a relatedness table (Step 1), the birth year of each participant in a pair is used to identify the older ("ID O") and younger ("ID Y") individual. Results are displayed sorted by the ID of the older individual first, and then that of the younger individual. If both share birth year, the individual originally identified as "ID 2" is arbitrarily considered older, such as in relationship ID (RID) 2 in the example. All individuals appearing in the pairs sharing an "ID O" value are then extracted into separate groups (Step 3), which are then used to construct a sparse matrix indicating which individuals belong to each group (Step 4; black background marks belonging, such as individual "H" appearing on groups G4 and G6 but not the rest). The cross-product of the sparse matrix in Step 5 then reveals which groups intersect, and its output can then be used to obtain clusters of groups through an intermediate graph representation (Step 6). Reconstructing which individuals belong to each group finally reveals the original family compositions (Step 7).
